# Human Papilloma Viruses and Breast Cancer

**DOI:** 10.3389/fonc.2015.00277

**Published:** 2015-12-16

**Authors:** James S. Lawson, Wendy K. Glenn, Daria Salyakina, Warick Delprado, Rosemary Clay, Annika Antonsson, Benjamin Heng, Shingo Miyauchi, Dinh D. Tran, Christopher C. Ngan, Louise Lutze-Mann, Noel J. Whitaker

**Affiliations:** ^1^School of Biotechnology and Biomolecular Science, University of New South Wales, Sydney, NSW, Australia; ^2^Center for Computational Science, University of Miami, Miami, FL, USA; ^3^Douglass Hanly Moir Pathology, Macquarie Park, NSW, Australia; ^4^QIMR Berghofer Medical Research Institute, Brisbane, QLD, Australia

**Keywords:** breast cancer, benign breast, human papilloma virus, HPV E7 proteins, retrospective cohort study

## Abstract

**Purpose:**

Human papillomaviruses (HPV) may have a role in some breast cancers. The purpose of this study is to fill important gaps in the evidence. These gaps are: (i) confirmation of the presence of high risk for cancer HPVs in breast cancers, (ii) evidence of HPV infections in benign breast tissues prior to the development of HPV-positive breast cancer in the same patients, (iii) evidence that HPVs are biologically active and not harmless passengers in breast cancer.

**Methods:**

RNA-seq data from The Cancer Genome Atlas (TCGA) was used to identify HPV RNA sequences in breast cancers. We also conducted a retrospective cohort study based on polymerase chain reaction (PCR) analyses to identify HPVs in archival specimens from Australian women with benign breast biopsies who later developed breast cancer. To assess whether HPVs in breast cancer were biologically active, the expression of the oncogenic protein HPV E7 was assessed by immunohistochemistry (IHC).

**Results:**

Thirty (3.5%) low-risk and 20 (2.3%) high-risk HPV types were identified in 855 breast cancers from the TCGA database. The high risk types were HPV 18 (48%), HPV 113 (24%), HPV 16 (10%), HPV 52 (10%). Data from the PCR cohort study indicated that HPV type 18 was the most common type identified in breast cancer specimens (55% of 40 breast cancer specimens) followed by HPV 16 (13%). The same HPV type was identified in both the benign and subsequent breast cancer in 15 patients. HPV E7 proteins were identified in 72% of benign breast specimens and 59% of invasive breast cancer specimens.

**Conclusion:**

There were four observations of particular interest: (i) confirmation by both NGS and PCR of the presence of high-risk HPV gene sequences in breast cancers, (ii) a correlation between high-risk HPV in benign breast specimens and subsequent HPV-positive breast cancer in the same patient, (iii) HPVs in breast cancer are likely to be biologically active (as shown by transcription of HPV DNA to RNA plus the expression of HPV E7 proteins), (iv) HPV oncogenic influences may occur early in the development of breast cancer.

## Introduction

High risk for cancer human papilloma viruses (HPVs) have been identified in breast cancers in 30 studies conducted in 17 countries and 4 continents (1, 2). HPV type 33 is the most prevalent (14%) but is mainly confined to Asian women, followed by HPV 18 (7%) and 16 (7%) (1, 2). In 10 case control studies, the prevalence of HPVs in breast cancers was significantly higher than in controls OR = 3.60 (1). Women with HPV-associated cervical neoplasia can later develop HPV-associated breast cancer (3, 4). However, several groups have not identified HPVs in breast cancer (5). All of the above studies have been based on polymerase chain reaction techniques (PCR).

High-risk HPVs have been identified in breast cancers by Next Generation Sequencing – NGS (also known as massive parallel sequencing)^1^. These studies have been based on The Cancer Genome Atlas (TCGA) database of 855 breast cancers. They demonstrate that high-risk HPVs are present in <2% of breast cancers and at very low viral loads. Recently, 1.3% of the TCGA specimens have been shown to be contaminated with HPV type 18 with the source probably HPV-positive HeLa laboratory cells (6). This contamination does not appear to affect the TCGA breast cancer specimens, which we have studied and reported in this current article (Cantalupo, 2015, personal communication). However, minor contamination cannot be excluded.

It is difficult to reconcile the data based on TCGA and analyzed by NGS, with the data based on Australian breast cancer specimens and analyzed by PCR. The TCGA data indicate a 2% prevalence of high-risk HPV-positive breast cancers compared to 60% of Australian breast cancers in this current study. However, of this 60% only approximately 40% had observable protein expression. Other studies based on PCR, such as the Ohba et al. study of breast cancer in Singapore residents (7), and prior studies of Australian breast cancers, have identified high-risk HPVs in from 25 to 100% of breast cancers (8, 9). The most likely explanation is the use of different viral identification techniques, namely, NGS as compared to PCR. NGS is a relatively new technique. Positive identification of HPV and other viruses is dependent on the recombination of fragmented gene sequences known as “reads.” There are no established conventions concerning the best length of the recombined “reads.” Therefore conclusions are in part, matters of opinion. While this may explain some of the different outcomes of NGS as compared to PCR, it is clear that the HPV viral load in breast cancer is extremely low as compared to cervical cancer.

While these data indicate that HPVs may have a possible role in some breast cancers, they may not have a major causal role. There are two reasons for this view: (i) in immunocompromised patients (due to either human immunodeficiency infections or post organ transplantation therapy), there is no increased prevalence of breast cancer, which is in contrast to the two- to sixfold increased prevalence of HPV associated cervical and head and neck cancer in these patients (10), and (ii) the HPV viral load in breast cancer is extremely low (11, 12).

However, it is important to determine whether high-risk HPVs have a causal role in even a small proportion of breast cancers. In addition to the epidemiologically based evidence outlined above, there is substantial but incomplete evidence, which indicates a potential role for HPVs in breast cancer. This evidence includes: (i) HPVs immortalize and transform human mammary epithelial cells (13), (ii) HPVs are located in breast cancer cell nuclei (8), (iii) HPV E6 oncoprotein has been identified in breast cancers (8), (iv) high-risk HPVs have been identified in breast cancer cell cultures (8), (v) HPV-positive breast cancer is more common in young as compared to older women, an observation which is compatible with sexual transmission of HPV among sexually active younger women (14, 15), (vi) approximately 16% of normal Australian women are serologically HPV type 16 positive (16), and (vii) HPV-positive koilocytes have been identified in breast cancers (17).

This evidence is incomplete. There is a need to determine (i) if high-risk HPVs are present in normal and benign breast tissues prior to the development of HPV-positive breast cancer and (ii) whether these HPVs are biologically active. These form the aims of this study.

## Materials and Methods

### Research Approaches

The research approach to the first aim – identification of HPVs – was to use a range of different methods for the identification of HPVs. These methods included Next Generation Sequencing (NGS), *in situ* and standard PCR, and immunohistochemistry. The approach to the second aim – identification of HPVs in benign non-malignant breast tissues prior to breast cancer – was conducted through a retrospective cohort study. The approach to the third aim – to determine the biological activity of HPVs in breast cancer – was to study HPV RNA sequences in breast cancers as indicators of transcription activity plus a study of HPV associated oncogenic protein expression.

### TCGA Next-Generation Sequencing Data and Bioinformatics Analysis

Whole transcriptome sequencing data for 855 (USA) Breast ­cancers were obtained through TCGA. Sequencing has been done using Illumina (Solexa, GAII) or SOLiD^TM^ technology. A detailed description of the TCGA data can be found on the following TCGA websites: http://cancergenome.nih.gov/, https://tcga-data.nci.nih.gov/tcga/. Transcriptome data (BAM files) generated by TCGA were analyzed in an automated fashion on a computational cluster hosted by the High-Performance Computing core at the Center for Computational Science, University of Miami^2^. All non-viral sequences were subtracted from NGS data as described in Salyakina and Tsinoremas (18). Reference genomes for all known HPV types obtained from PaVE database^3^ were used for HPV screening. Even the single short read 48 nt or longer aligning to the viral reference was considered as successful detection, if following BLAST analysis versus the NCBI nucleotide collection confirmed sequence similarity with the target over 98%. Sequences aligning to multiple organisms, known artificial (vector) sequences, or low complexity sequences were considered false positive and removed.

The outcomes for HPV 18 were compared to the outcomes of the Larsson lab for the same TCGA breast cancer specimens.

APOBEC gene family mutations and expression data for TCGA cohort were available for 850 out of 855 analyzed samples from http://www.cbioportal.org. The Fisher exact test was employed to test possible association of HPV status with APOBEC genes expression or mutations.

Clinical data was available for 836 out of 855 patients from the TCGA portal.

These analyses were conducted by Daria Salyakina at the Center for Computational Science, University of Miami, Coral Gables, FL, USA.

### Retrospective Cohort Study

We conducted a retrospective cohort study to identify HPV sequences in benign breast biopsy tissues in women who later developed HPV-positive breast cancer. This is a practical and low-cost method of achieving sound outcomes. If the HPV DNA was of the same type and sequence in both the prior benign breast tissues as in the subsequent breast cancer tissues from the same patient this potentially provides evidence of prior HPV breast infection. All the specimens were from women residing in Australia. We reviewed approximately 4,000 pathology reports of breast cancer and identified 41 patients who had benign breast biopsies 1–11 years prior to developing breast cancer. The pathology reports of associated archival formalin fixed specimens from these patients were identified and collected from an Australian pathology service (Douglas Hanly Moir Pathology). The average age of the patients with benign breast conditions was 50.5 years and subsequent cancer 56.1 years.

We used normal breast specimens from 21 patients who had cosmetic breast surgery as a comparative group. The average age of these donors was 35.7 years. These patients were much younger than the patients who developed breast cancer, and therefore are not an exact control group. None of these patients had developed breast cancer 10 years after the initial surgery.

### Identification of HPV Gene Sequences by Polymerase Chain Reaction

*In situ* PCR, semi-nested PCR, and real-time PCR were used for the detection of HPV. All PCR products were sequenced to help identify any contamination. Although *in situ* PCR can produce false positive outcomes, use of this method can add to the validity of results based on semi-nested and real time PCR. The detailed methods are presented in Supplementary Materials and Methods.

#### Preparation of Genomic DNA

For standard and real-time PCR, the methods were as described in Steinau et al. (19). The primers used were β-actin 5′ *forward* (5′-CTTCTGCCGTTTTCCGTAGG-3′) and β-actin 3′ *reverse* (5′-TGGGATGGGGAGTCTGTTCA-3′).

#### Standard PCR

The primers for semi-nested PCR My11 (5′-GCACAGGGYCAYAAYAATGG-3′) to modified GP6 (5′-AATCATATTCCTCMMCATGTC-3′). The second round was Gp5+ (5′-TATTTGTTACTGTKGTWGATAC-3′) to Gp6+. These primers were degenerate for HPV16 and 18, but were also capable of bringing up types 3, 11, 12, 45, 58, 73, and 75.

#### Real-Time PCR

The HPV L1 gene in gDNA samples was amplified using a real-time PCR machine (Rotor Gene Q, QIAGEN).

##### Sequencing the PCR Products and Identification of HPV Types

The HPV PCR products from GP5 to Gp6 were sequenced to determine the HPV type. The HPV genotypes were identified by BLAST via the US National Center for Biotechnology Information.

#### *In situ* PCR

These analyses were conducted as described in Heng et al. (8). *In situ* PCR is less susceptible to contamination and has the important advantage of localizing the specific genetic material at the cellular level.

The identification by PCR of HPV in benign non-malignant breast or breast cancer specimens was considered as positive if two or more of the following outcomes in the same specimen were observed: (i) HPV DNA sequences identified by standard PCR and/or real time PCR, (ii) HPV positive *in situ* PCR.

#### Confirmatory PCR Studies

DNA extracts from six selected (for positive HPV) benign and breast cancer specimens were independently analyzed by the Antonsson group at the QIMR Berghofer Medical Research Institute, Brisbane, for the presence of HPV DNA using their previously published methods (20).

### HPV Biological Activity

An assessment of HPV biological and oncogenic activity was conducted by (i) the identification of HPV RNA sequences, which are an indication of HPV transcription activity (ii) the identification of HPV E7 proteins, (iii) the high expression of p16 protein, (iv) high expression of ER, and (v) inhibition of p53 protein expression.

Antibodies that are specific for HPV E7 proteins, have recently been developed (21). These antibodies (Cervimax) were used in this study. The specificity of these antibodies has been demonstrated experimentally and by epidemiological studies (21). The HPV E7 antibody reacts with a wide range of HPV types including high risk for cancer HPV 16 and 18. HPV E7 antibodies have not been previously used on breast tissues.

#### p16

p16 contributes to regulation of the cell cycle. There is substantial evidence that indicates that p16 expression is associated with HPV biological activity (22). High expression of p16 as a surrogate for HPV E7 activity is a sound indication of transcriptionally active HPV (23). In breast tissues, negative or low expression of p16 is present in normal ductal epithelial tissues with a progressive increase of p16 expression in benign and malignant breast lesions (24–26). The situation is complicated by Epstein–Barr virus (EBV), which blocks the expression of p16 (27).

#### p53

The inhibition of expression of the cell death related p53 protein by HPV E6 protein is a well-documented mechanism for HPV oncogenesis. p53 expression is inhibited in HPV 16-positive breast cancer (28).

#### Estrogen Recptor

Estrogens have an essential role in breast cancer and high ER expression is a feature of most breast cancers (29). Recently, high ER expression has been associated with HPV-positive breast cancers (30).

#### HER 2 (ErbB-2)

The HER 2 receptor co-operates with HPV E6 and E7 oncoproteins in breast oncogenesis (31).

### Immunohistochemistry

Standard manual IHC methods were used with the omission of the antigen retrieval step for the identification of HPV E7 protein expression. The antibodies were anti-HPV E7 monoclonal “Cervimax” – Valdospan GmbH, Austria. Positive controls for the E7 antibody were the Hela (HPV18) cell line and cervical tissues that were positive by PCR and sequencing.

Immunohistochemistry for estrogen receptor (ER), progesterone receptor (PR), and human epidermal growth factor receptor 2 (HER 2) were previously performed as part of the standard assessment of breast cancer specimens. For benign breast specimens, the same assessments were conducted as part of this current project.

ER, PR, p16, and p53 proteins are expressed in cell nuclei. HER 2 protein is expressed in cell cytoplasm and membranes. HPV types 16 and 18 E6 protein is expressed in both cell nuclei and cytoplasm. Anatomical pathologists from Douglass Hanly Moir – Pathology conducted the routine assessment of ER, PR, and HER in breast cancer specimens. Two independent observers (James S. Lawson and Wendy K. Glenn) used the same methods for the assessment of ER, PR, and HER in the benign breast specimens. The staining of breast tissues during immunohistochemistry procedures is heterogenous with staining frequently confined to only part of a tissue section. The assessments were based on those tissue sections with positive staining cells. The outcomes were assessed by the intensity of staining on a scale of 0 and 1–3. Positive (HPV-positive cervical cancer specimen) and negative (antibody omitted) controls were used for each batch of specimens.

In breast cancer specimens, IHC staining for p16 appears to differ from the patterns commonly seen in cervical neoplasia. p16 expression in both non-invasive and invasive breast cancer specimens are most commonly seen in clusters of cancer cells with no staining in other parts of the cancer specimen (23). For this reason, we have adopted the same methods of assessment as outlined above for ER and PR but confined to assessments of percentages of positive and intensity of staining cancer cells in the clusters. We have adopted the same approach to assessment of p53, which also commonly stains in clusters in breast cancer specimens.

### Statistics

Associations between HPV identification and various breast specimens were tested for correlations using the SPSS statistical package. The tests were all two-sided, and statistical significance was defined as *p* ≤ 0.05. The numbers in this study are small. Accordingly, it is necessary to be cautious despite some of the results reaching statistical significance. The specific statistical tests used are listed with the different results sections.

## Results

### Identification of HPV Gene Sequences in the TCGA Breast Cancers Cohort

Whole transcriptome data identified 30 (3.5%) tumors with low-risk and 20 (2.3%) with high-risk HPV types in 855 breast cancers from the TCGA cohort (Table S1 in Supplementary Material). The high risk types that were identified in the TCGA cohort were HPV 18 (50%), HPV 113 (20%), HPV 16 (10%), HPV 52 (10%) plus one identification of HPVs 31 and 35 (5% each) (Table 1). Three specimens had two HPV types.

**Table 1:** **HPV types in TCGA breast cancers – next generation sequencing**.

**High-risk HPVs *n* = 20**	
HPV 16 – 2 (10%)	HPV E1, E2, E6, E7, and L1 identified
HPV 18 – 10 (50%)	HPV E1, E6, and E7 identified
HPV 31 – 1 (5%)	HPV E7 identified
HPV 35 – 1 (5%)	HPV E7 identified
HPV 52 – 2 (10%)	HPV E2, E4, E5, and E7 identified
**Low-risk HPVs *n* = 30**	
HPV 4 – 3 (10%)	
HPV 17 – 3 (10%)	
HPV 22 – 2 (7%)	
HPV 38 – 2 (7%)	
HPV 49 – 3 (10%)	
HPV 65 – 2 (7%)	
HPV 113 – 4 (20%)	HPV L1, L2, E7 identified
HPVs 1, 4, 5, 10, 15, 17, 19, 22, 23, 24, 38, 49, 63, 65, 80, 93, 104, 110, 124, 132, 137 – one identification each.	

*Low and high risk types of HPV sequences were identified in 50 of 855 TCGA breast cancers. E5, E6, and E7 oncogenes were identified*.

As outlined in the Section “Introduction” of this report, 1.3% of the TCGA specimens have been shown to be contaminated with HPV type 18. The source of contamination was probably HPV-positive HeLa laboratory cells (6). This contamination does not appear to affect the TCGA breast cancer specimens that we have studied and report in this current article (Cantalupo, 2015, personal communication). However, minor contamination cannot be excluded.

When considered together, the HPV nucleotides that have been identified in the 50 breast cancer specimens range from position 109 to 6589 on the HPV 18 complete genome, which has approximately 7,800 nt.

**Figure 1:**
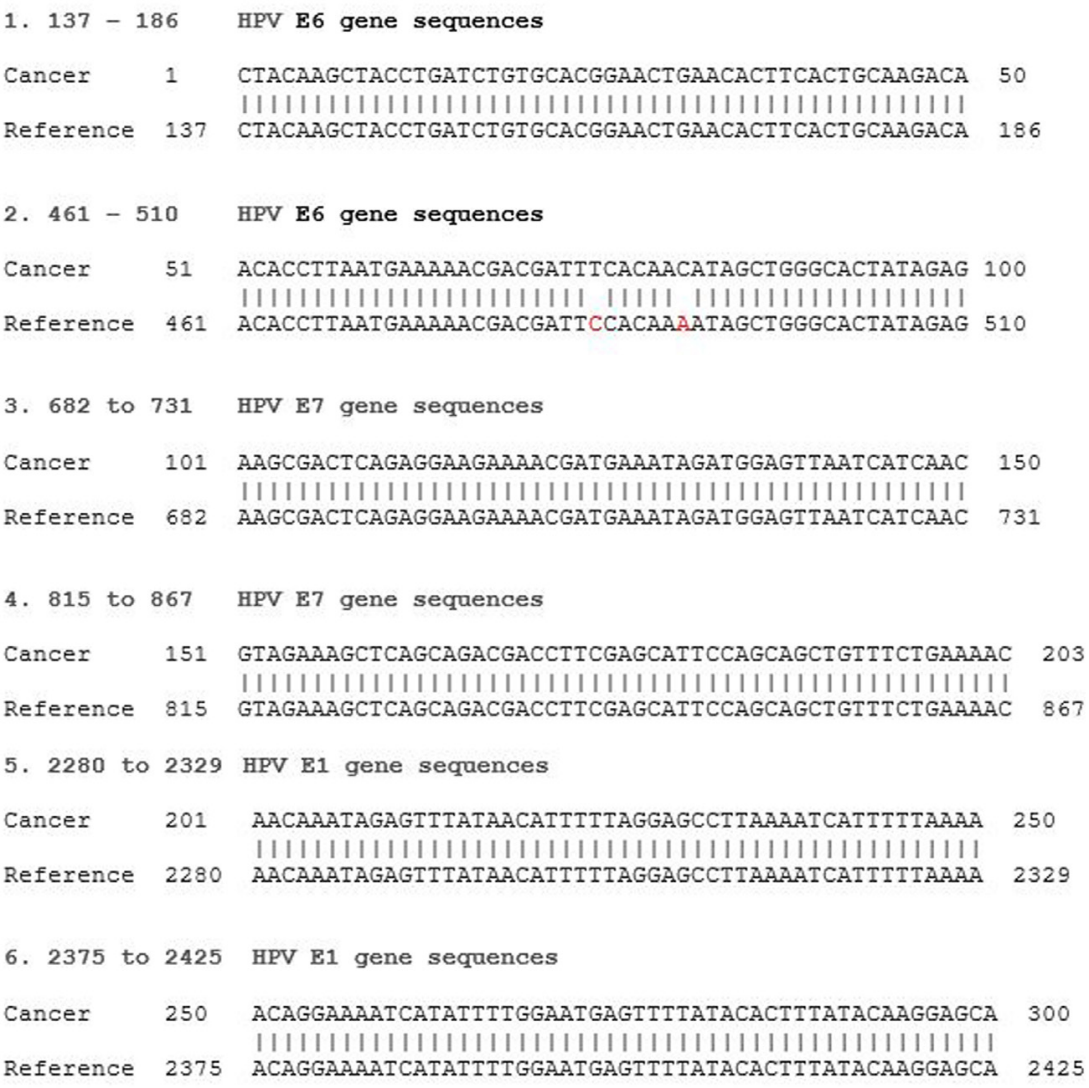
**HPV type 18 E6 and E7 RNA gene sequence identified in an invasive breast cancer specimen by Next Generation Sequencing**. Invasive breast cancer specimen 36 TCGAAR-A24P-01A-11R-A169-07 (see Table S1 in Supplementary Material) sequence compared to reference HPV 18 KC470228. E6 gene sequences reference 105-581, E7 590-907, E1 914-2887. The two point mutations at reference 484 and 490 have been observed in reference HPV 18 KC470209.1.

The majority of breast tumors had extremely low levels of viral expression with one or two short reads identified resulting in 50–100 nt long unique HPV fragments. Only four of the tumors showed longer continuous sequences of 300 nt or more. All identified HPV sequences had high and specific homology to reference HPV genomes. Short reads were randomly spread over thousands of nucleotides of the viral genome with large gaps between them due to the low viral load. An example of sequence reads can be seen in Figure 1.

The outcomes for HPV type 18 were identical to those of the Larsson lab.

HPV E7 gene sequences were identified in each of the high-risk HPV groups of breast cancers. HPV E7 oncoproteins are involved in oncogenesis. HPV E6 and E7 protein gene sequences by NGS in breast cancer are shown in Figure 1. The homology between the identified viral and reference sequences was greater than 95%. These HPV RNA sequences indicate that this virus has the capacity to express HPV E6 and E7 proteins, which interact with p53 and Rb to cause malignant transformation.

Associations between HPV and APOBEC could not be detected in the TCGA cohort with respect to either mutations or high/low APOBEC expression levels (*p* > 0.05).

### Identification by PCR of HPV Gene Sequences in Australian Benign and Subsequent Breast Cancers

As shown in Table 2 and Table S2 in Supplementary Material, fifteen (65%) of the HPV-positive specimens were of the same type in both the benign and subsequent breast cancer (p = 0.001).

**Table 2:** **High risk HPV sequences in benign breast and subsequent invasive ductal carcinoma (IDC) breast cancer specimens in the same patients**.

HPV sequences	Same HPV type: benign/cancer	Nil HPV benign HPV- positive cancer	HPV-positive benign Nil HPV cancer
HPV 16		1	1
HPV 16/18	2		
HPV 18	13	3	3
HPV 58		1	1
Nil HPV	10		

*Fifteen (65%) of the HPV-positive specimens were of the same type in both the benign and subsequent breast cancer (p = 0.001)*.

Consistent variations in the HPV sequences of both the benign and subsequent IDC breast cancers in the same patients is suggestive that the same HPV infection is involved in both sets of breast tissues. HPV 18 sequences were identified in five of seven ductal carcinoma *in situ* (DCIS) breast cancer specimens. HPV 18 was identified in three of seven benign and subsequent DCIS specimens. The PCR products that were detected and sequenced are from the L1 region of the HPV genome.

HPV type 18 was the most common type identified in breast cancer specimens (55% of 40 IDC and DCIS breast cancer specimens) followed by HPV 16 (13%) and uncommonly HPV high-risk types 45, 58, and 73. Low-risk HPV types 3, 78, and 124 plus three different new HPV types were also identified. There were mixed HPV types in both prior benign and later breast cancer specimens. HPV viruses were not identified in 7 of 40 (17.5%) of sets of both benign and subsequent breast cancer specimens. Sequences from one benign and three invasive breast cancer specimens contained novel HPV sequences whose identity was the same as that reported from a Sydney renal transplant patient (AF019978).

The details of these findings are shown in Table S2 in Supplementary Material.

**Figure 2:**
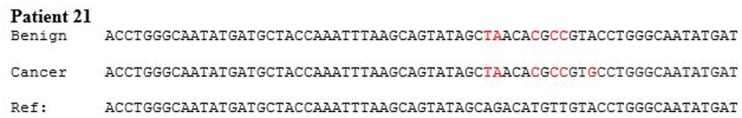
**HPV type 18 DNA sequences in benign breast and subsequent breast cancer**. There are identical sequence variations of a PCR product Gp5+ to Gp6+ in both the benign and later breast cancer in patient 21. (The sequence has been abbreviated to include the differences). This indicates it is the same virus and differs from the reference sequences based on HeLa cervical cell culture.

**Figure 3:**
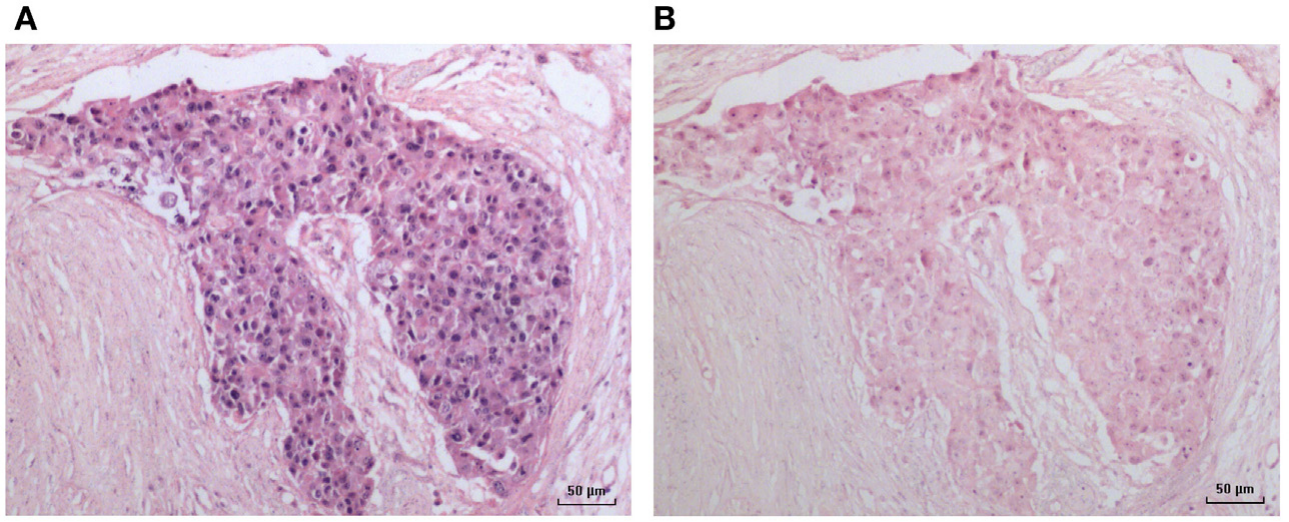
**HPV by *in situ* PCR in a breast ductal carcinoma *in situ* specimen**. **(A)** HPV; **(B)** Negative (no primers) control.

HPV type 18 DNA sequences in benign and subsequent breast cancer in a typical patient are shown in Figure 2. HPV sequences in a breast DCIS specimen is shown in Figure 3. As demonstrated in Figure 2, there are identical sequence variations of a PCR product Gp5+ to Gp6+ in both the benign and later breast cancer. These variations differ from the reference sequences based on HeLa cervical cell cultures and suggest that HPV infection of benign breast tissues has later become the same HPV virus-positive invasive breast cancer. In other HPV 18-positive sets of benign and later breast cancers, the sequence patterns were identical in the benign breast, subsequent breast cancer, and reference HeLa sequences.

An HPV 18-positive mixed DCIS and invasive ductal carcinoma of the breast identified by *in situ* PCR is shown in Figure 3. This image is typical of other images of invasive and non-invasive breast cancers identified by *in situ* PCR.

The identification of different, new, and novel HPV types in both the benign and breast cancer specimens indicates that false positive outcomes due to contamination during PCR analyses are unlikely.

The difference in prevalence of HPV sequences between normal breast and benign breast is significant (*p* = 0.001) and between normal breast and breast cancer is significant (*p* = 0.001) (Table 3).

**Table 3:** **Identification by PCR of high risk for cancer HPVs in normal breast (source cosmetic surgery), benign breast and breast cancer (same patients with prior benign breast and subsequent breast cancer)**.

Tissue type	High risk HPV sequences
Normal breast *n* = 21	6 (29%)
Benign breast *n* = 40	22 (55%)
Breast cancer *n* = 40	27 (66%)

*The difference in prevalence of HPV sequences between normal breast and benign breast is significant (*p* = 0.001) and between normal breast and breast cancer is significant (*p* = 0.001)*.

### Confirmation of Data Based on PCR

DNA extracts from six HPV-positive benign and breast cancer (extracted and analyzed at the University of New South Wales laboratories) patients were independently analyzed at the Antonsson laboratories in Brisbane, QLD, Australia. HPV types 16 and 18 were identified in 2 of 6 specimens. The identification of different HPV types indicates that contamination is unlikely.

### Indicators of HPV Biological Activity and Oncogenic Influences

#### Identification of RNA by Next-Generation Sequencing

The identification of DNA viruses in cancer tissues by NGS involves recognition of RNAs. DNA viruses must be biologically active to transcribe into RNAs. Therefore, the outcomes of NGS in this investigation provide evidence that the high-risk HPVs identified are biologically active. In addition, the identification of HPV E6 and E7 genes in these high-risk HPV sequences is an indication that they are capable of expressing HPV E6 and E7 oncoproteins.

#### Immunohistochemistry for HPV E7

The data are shown in Table 4 and in detail in Table S2 in Supplementary Material. The prevalence of HPV E7-positive protein expression is similar in both HPV-positive and HPV-negative benign breast specimens. The prevalence of HPV E7-positive protein expression is significantly higher in HPV DNA sequence-positive as compared to HPVsequence-negative breast cancer specimens (Pearson chi-square *p* = 0.007).

**Table 4:** **HPV E7 protein expression in high risk HPV-positive and -negative benign breast and invasive ductal carcinoma (IDC) and ductal carcinoma *in situ* (DCIS) breast cancer**.

HPV DNA sequences (PCR)	HPV E7 positive (immunohistochemistry)	HPV E7 negative (immunohistochemistry)
**Benign breast**		
HPV positive *n* = 20	13 (65%)	7 (35%)
HPV negative *n* = 11	8 (73%)	3 (27%)
**IDC and dcis breast cancer**		
HPV positive *n* = 21	16 (76%)	5 (24%)
HPV negative *n* = 9	2 (22%)	7 (78%)

*The prevalence of HPV E7-positive protein expression is similar in both HPV-positive and HPV-negative benign breast specimens*.

*The prevalence of HPV E7-positive protein expression is significantly higher in HPV DNA sequence positive as compared to HPV-negative breast cancer specimens (Pearson chi-square *p* = 0.007)*.

**Figure 4:**
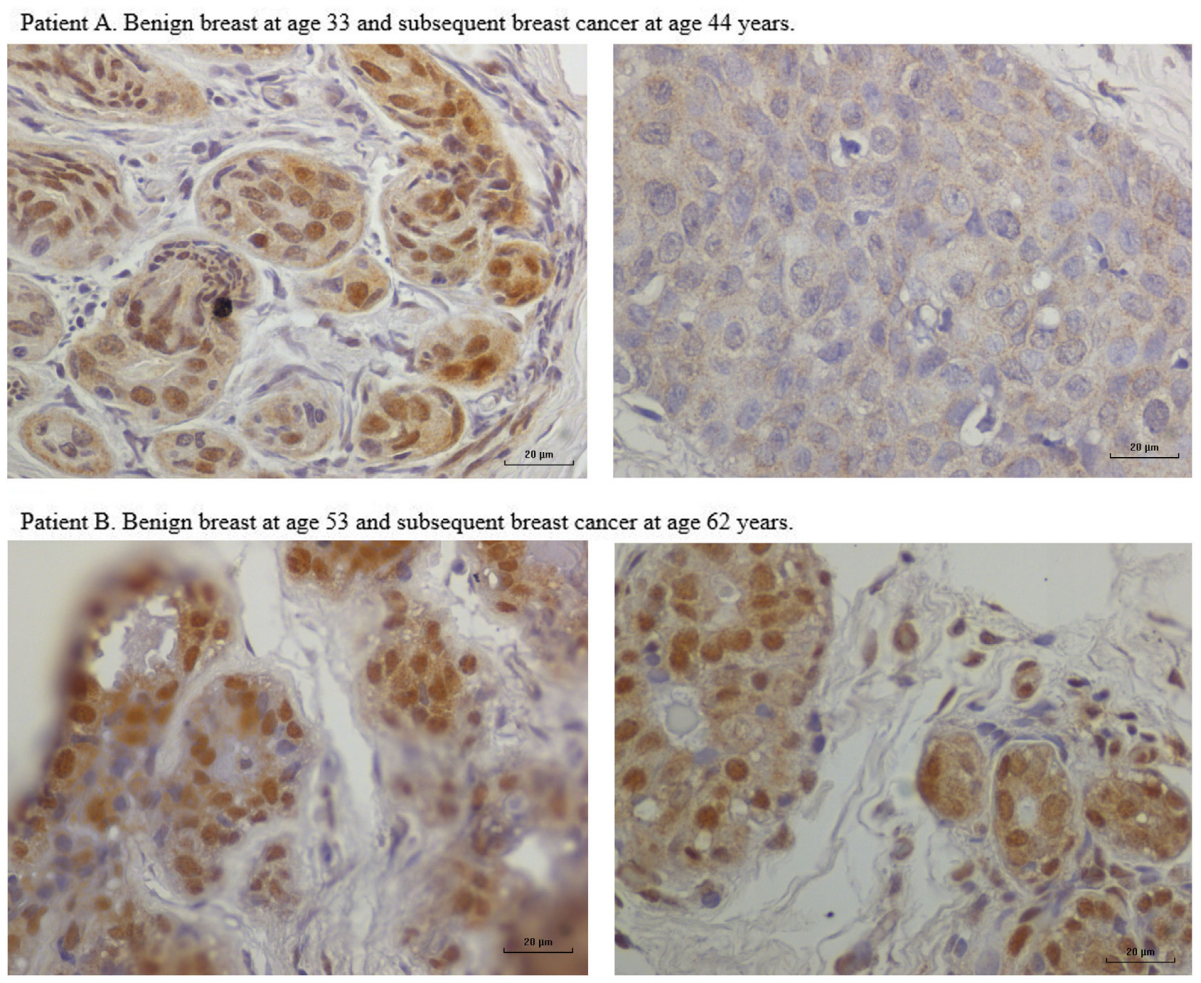
**HPV E7 protein expression in HPV 18-positive benign and subsequent HPV 18-positive breast cancer demonstrated by immunohistochemistry**. Patient A. Benign breast specimen from a patient at age 33 years and subsequent invasive breast cancer in the same patient aged 44 years. HPV E7 protein brown staining is positive, blue staining is negative. There is strong HPV E7 protein expression in the nuclei of benign specimen. There is no HPV E7 protein expression in the subsequent invasive breast cancer specimen from the same patient. These observations are compatible with HPV having a role early in breast oncogenesis. Patient B. Benign breast specimen from a patient at age 53 and subsequent breast cancer in the same patient aged 62 years. There is strong HPV E7 protein staining in both the benign and subsequent breast cancer.

**Figure 5:**
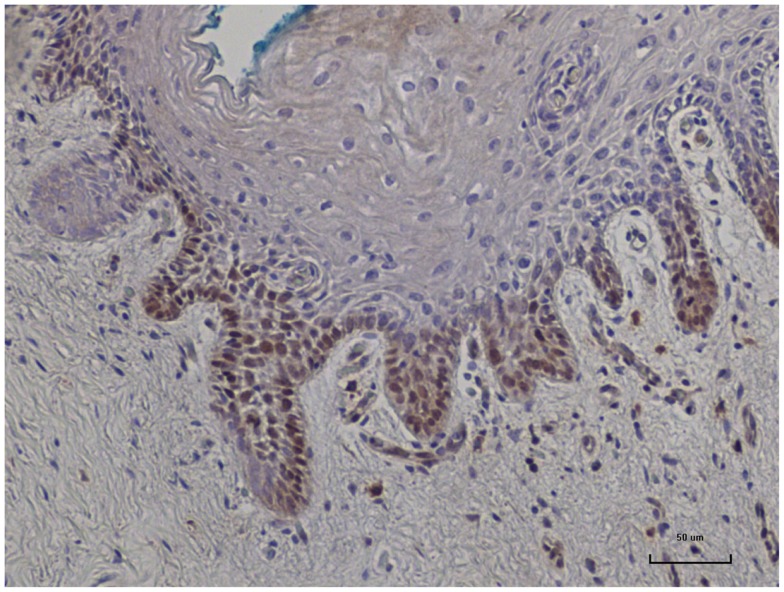
**HPV E7 protein expression identified in HPV type 18 positive cervical intraepithelial neoplasia (CIN 1)**. This is a positive control for Cervimax antibodies used in the immunohistochemistry analyses.

In nine sets of specimens, there was strong HPV E7 expression in the benign breast specimens but little or no HPV E7 staining in the subsequent breast cancer that developed later in the same patient. This is illustrated in Patient A in Figure 4 in which there is high HPV E7 protein expression in the cell nuclei of the benign breast specimen but no expression in the nuclei of the subsequent breast cancer specimens. This pattern was not observed in four sets of specimens, in which the HPV E7 protein expression was higher in the cancer than the prior benign breast cell nuclei. This different pattern of HPV E7 protein expression is shown in Patient B in Figure 4. The implication is that the oncogenic influence of HPVs may be early in step by step oncogenesis in some patients. This will be considered in detail in the Section “Discussion.” The positive control for HPV E7 protein expression – HPV type 18 cervical intraepithelial neoplasia (CIN 1), is shown in Figure 5.

There were no correlations between p16, p53, ER, PR, and HER2 protein expression in benign breast biopsy tissues and subsequent HPV-positive breast cancer (Table S2 in Supplementary Material). There were no correlations between the identification of HPVs and the presence of proliferative breast epithelial cells in benign breast biopsy tissues.

p16 proteins were highly expressed (2 or 3 on a scale of 0–3 staining intensity) in 12 benign and 11 invasive breast cancers in which high-risk HPVs had been identified (Table S2 in Supplementary Material). However, similarly high p16 expression was present in four benign and five invasive breast cancers in which HPV sequences were not identified.

##### Estrogen receptor protein expression

The expression of ER was very high in normal, benign, and subsequent breast cancer (Table S2 in Supplementary Material) whether or not HPV was identified. Therefore, HPV influences cannot be implied from these data.

##### HER2 protein expression

There was no correlation between the identification of HPV sequences and HER2 protein expression in either benign or later breast cancer.

### Left or Right Breast Cancer Following Prior Benign Breast Conditions

In the same patients, 49% of the breast cancers developed in the same side as the benign breast condition and 51% developed in the opposite side breast. There was no association between HPV-positive breast cancers and whether the side of breast was the same as the prior HPV-positive benign condition. These observations suggest that if HPVs are oncogenic in breast cancer, the prior HPV infection and oncogenic influences may affect both breasts but only one develops breast cancer.

## Discussion

These investigations indicate (i) data based on the TCGA by Next-Generation Sequencing (NGS) confirms the identification of high-risk HPVs in approximately 2% of breast cancers, (ii) that high-risk HPV sequences of the same type identified by PCR are present in 37% of benign non-malignant breast and subsequent non-invasive and invasive breast cancers in the same Australian patients, and (iii) high-risk HPVs in breast cancer are biologically active as indicated by HPV E7 protein expression and the transcription of HPV DNA to RNA. In addition, the observation that HPV E7 protein may be highly expressed in the nuclei of benign breast cells but not expressed in the nuclei of subsequent breast cancer cells suggests HPV biological activity in breast cancer may be an early phenomena. This differs from cervical cancer, which usually requires the continuing presence of active HPVs for oncogenic progression.

In this study, HPV type 18 was the most frequently identified type. In each of the previous studies of HPV in Australian breast and prostate cancers, HPV type 18 has been the most common type (8, 9, 32). Types of high-risk HPVs vary in different populations. For example, HPV type 58 is common in Chinese breast cancers and HPV types 33 and 35 are the most common in Syrian breast cancers (33). HPV type 18 was the most common type identified by the Larsson Lab in the TCGA breast cancer data bank, which contains predominently US breast cancers. HPV type 16 was the most common type recently identified in breast cancers in Egyptian patients (34).

### Validity of the Findings

The use of a range of techniques, namely, NGS, *in situ* PCR, nested PCR, real-time PCR, and immunohistochemistry, substantially adds to the validity to the outcomes. However, the identification of HPVs by NGS is much lower than their identification by PCR techniques. The high number of short “reads” by NGS can be regarded as an indication of possible false positives; however, the very high specificity and homology to HPV reference sequences suggests the outcomes are valid. The findings based on NGS confirm the extremely low HPV viral load in breast cancer.

The finding by both NGS and PCR of a variety of HPV types, suggests these HPV sequences were derived from patient samples and not contaminants. With respect to outcomes, based on NGS, there does not appear to be contamination (Cantalupo, 2015, personal communication). However, as previously indicated, minor contamination cannot be excluded. The identical identification by NGS of HPV 18 in the same TCGA breast cancers by the Larsson lab and in this current study adds to the validity of the NGS results. The outcomes of the investigations based on PCR are in agreement with the recent results of Ohba et al (7) who identified high-risk HPVs in 20 to 100% of breast cancers in different ethnic groups in Singapore. They used novel DNA chip technology for the identification of HPVs.

The HPV E7 gene sequences identified by NGS add validity to the outcomes based on immunohistochemistry.

### Identification of High-Risk HPVs in Benign Non-Malignant Breast and Subsequent Breast Cancer

The much higher prevalence of HPV sequences in both the benign non-malignant breast and later breast cancer as compared with their prevalence in normal breast tissues from cosmetic surgery is striking. It is particularly noteworthy that HPV type 18 sequences with identical variations from the HeLa reference sequence were identified in benign breast tissues and 1–11 years later in breast cancer in the same patients. This suggests that it is probably the same virus in both the benign and subsequent breast cancer that developed later in the same patient. This finding adds to the Hill (35) criteria for causation.

HPV type 18 is the most common HPV type in breast cancer among Australian women (8, 9). HPV type 18 is also tropic to glandular cervical epithelial cells (36). The high (55%) proportion of HPV type 18 identified by PCR is very similar to the proportion of HPV type 18 identified in adenocarcinoma of the cervix (36). It is therefore of considerable interest that HPV 18 was by far the most common HPV type identified by NGS in the TCGA breast cancers.

### Conflicting Data

It is difficult to reconcile the data based on TCGA and analyzed by NGS, with the data based on Australian breast cancer specimens and analyzed by PCR. The TCGA data indicates a 2% prevalence of high-risk HPV-positive breast cancers compared to 60% of Australian breast cancers in this current study. Other studies based on PCR, such as the Ohba et al. study of breast cancer in Singapore residents (7), and prior studies of Australian breast cancers, have identified high-risk HPVs in from 25 to 100% of breast cancers (8, 9). The most likely explanation is the use of different viral identification techniques, namely, NGS as compared to PCR. An additional explanation is that only about 40% of the 60% of Australian breast cancers that were positive for high-risk HPV sequences had observable HPV protein expression. As the TCGA data is based on protein expression, this may explain part of those differences.

However, this does not explain the two- to six-fold higher prevalence of other HPV associated cancers, such as cervical cancer, in immunocompromised patients, as compared to immunocompromised patients with breast cancer (10). There are several possible explanations. One, HPVs may be present in breast cancers but are mostly not oncogenic, and are therefore unaffected by immunosuppression. Two, the oncogenic mechanisms of HPVs in breast cancer may differ from those in cervical cancer and may not be affected by immunosuppression. In our view, the second explanation is the most likely.

The mechanisms of HPV oncogenesis in cervical cancer are well established and include the influences of HPV E6 and E7 oncoproteins, which work in concert to disrupt cell-cycle regulation, inhibit apoptosis, and stimulate cell cycle progression by binding and inhibiting the p53 and p110RB tumor suppressor genes (28). In most instances, continued presence of HPV is required for cervical oncogenesis. In addition, HPV E5 and E6 act early in transformation (before integration) and are known to disrupt cytokeratin causing perinuclear cytoplasmic clearing and nuclear enlargement, which leads to the appearance of koilocytes (cells with halos surrounding the nuclei) (28).

There is experimental evidence that indicates there are different HPV oncogenic mechanisms in breast cancer as compared to cervical cancer. Much of this evidence has been developed by Ohba et al. in Singapore (7). They have demonstrated (i) that breast cancer cells with high ER protein are significantly correlated with high HPV infections (ii) that HPVs appear to activate APOBEC3B enzymes leading to genomic instability – abnormal APOBEC3B protein expression has been associated with increased breast cancer risk (37, 38). These oncogenic influences occur in the early stages of multi-step, multi-factorial breast carcinogenesis.

In this current study, we have observed there may be higher levels of HPV E7 protein expression in the nuclei of benign breast glandular cells as compared to HPV E7 expression in the nuclei of subsequent breast cancer cells in the same patients. This observation supports the hypothesis that HPVs may be involved in the early stages of breast oncogenesis. This may be the HPV “hit and run” phenomenon previously described by others (39).

Overall, this evidence offers a plausible explanation for the lack of influence of immunosuppression in HPV associated breast cancer.

### Indicators of HPV Biological Activity and Oncogenic Influences

The outcomes of NGS in TCGA data in this investigation provide suggestive evidence that the high-risk HPVs identified are biologically active. This is demonstrated by the recognition of HPV RNAs by NGS. This is because HPVs are DNA viruses that generate RNA transcripts for the expression of oncogenic proteins.

There was a significant correlation (*r* = 0.603, *p* = 0.004) between positive and negative HPV sequence identification and HPV E7 protein expression in normal (cosmetic surgery) breast tissues. The implication is that HPV E7 protein is a likely indicator of HPV biological activity in breast tissues. However, 10 years later, none of the women who had HPV-positive normal breast tissues had developed breast cancer. Therefore, the identification of HPV sequences and HPV E7 protein expression in benign breast tissues does not necessarily lead to subsequent breast cancer.

The inhibited expression of p53 protein in benign non-malignant breast and breast cancer observed in this study parallels the prior observation by Hennig et al (3). The inhibition of expression of the cell death related p53 protein by HPV E6 protein is a well-documented mechanism for HPV oncogenesis (28).

The role of p16 in breast cancer is not clear. In this current study, there were no significant differences in p16 protein expression as assessed by IHC between normal, benign, and malignant breast tissues. In addition, there were no significant correlations between high p16 protein expression and triple negative breast cancer as has been observed in other studies (40, 41). High p16 protein expression has been associated with HPV infections in breast, cervical, and other cancers (40, 41). However, in these and other studies of p16 expression in breast cancer, normal breast controls were not used, and therefore is not possible draw any conclusions from our current data.

### HPV Transmission

The mode of transmission of HPV to the breast is not known. However, it is possible that HPVs are transmitted to the genital tract during sexual activities and later transmitted by white blood cells throughout the body. including the breasts. This hypothesis is based on the young age of women with HPV-positive breast cancer. These young women are sexually active and have a high incidence of HPV cervical infections (42). In addition, there have been repeated identification of high-risk HPVs in white blood cells (43).

### Breast Cancer is Hormone Dependent

The presence of a hormone influenced virus in breast cancers is of special interest. The levels of ER expression in normal, benign, and breast cancer specimens in this study were very high. High ER expression in association with HPV-related breast cancer was also observed by Ohba (7). Estrogens synergize with high-risk HPV E6 and E7 oncogenes to cause human cervical cancer (44). In addition, the regulatory region of HPV 16 genomic DNA contains sequences that are responsive to glucocorticoid hormones. The HPV18 regulatory region contains one functional GRE sequence that interacts with the glucocorticoid receptor and confers hormonal activation to HPV18 promoter (45).

### Benign Breast Disease as Precancerous

These data indicate the marked differences between normal breast and benign breast tissues. These differences include the significantly higher prevalence of HPV sequences and proliferation of epithelial cells in benign as compared to normal breast tissues. In addition, there is a significant correlation between benign and later breast cancer in the same patient for the identification of HPV gene sequences and abnormal biomarker expression, including HPV E7 protein expression. While it is well documented that “benign” breast hyperplasia (glandular cell proliferation) leads to a fourfold increased risk of breast cancer, the observations in this current study, suggest that non-proliferative benign breast disease may also be precancerous (46, 47).

### Left or Right Breast Cancer Following Prior Benign Breast Conditions

The observation that there was no association between the side of HPV-positive benign breast conditions and the side of later HPV-positive breast cancer is of considerable interest. It may be that prior HPV infection and oncogenic influences may affect both breasts but only one develops breast cancer. However, there is no evidence from this current study to support this notion. The development of breast cancer in both breasts either at the same or at different times occurs in women with and without a genetic predisposition to develop breast cancer (48). The risk of bilateral breast cancer at different times is higher among young women (48). This is compatible with an HPV infectious cause of the cancer.

## Conclusion

In this current study, there were four observations of particular interest: (i) confirmation by both NGS and a range of PCR techniques, of the presence of high-risk HPV gene sequences in breast cancers, (ii) there is a correlation between high-risk HPV gene sequences in benign breast biopsy specimens and subsequent HPV-positive breast cancer in the same patient, which is an indication of prior HPV infection before the development of HPV-positive breast cancer, (iii) that HPVs in breast cancer are likely to be biologically active (as shown by transcription of HPV DNA to RNA plus the expression of HPV E7 proteins), and (iv) that HPV oncogenic influences may occur early in the step by step development of breast cancer.

## Ethics Statement

This project has formal ethics approval by the University of New South Wales Human Research Ethics Committee – number HREC HC11421. Participants gave written informed consent to participate in this study.

Ethics approvals for the follow up of women who donated normal breast tissues was given by the New South Wales Population and Health Services Research Ethics Committee – number 2009/12/203. This Ethics committee waived the need for consent. The reasons for waiving consent were (i) the specimens were archival having been collected in 1999, 2000 and 2001, (ii) all specimens were “de-identified” to the research group and (iii) retrospective approaches to donors, all of whom had cosmetic surgery, may have caused unnecessary anxiety.

## Author Contributions

Conceived, designed, and co-ordinated the project: JSL. Conducted laboratory analyses and interpreted the data: WG, AA, BH, SM, DT, CN, LL-M, NW. Conducted the Next Generation Sequencing and interpreted the data DS. Identified the patients and collected the archival specimens: RC, JL. Identified patients, conducted histopathology WD. Wrote the manuscript: JL, WG, NW.

## Conflict of Interest Statement

The authors declare that the research was conducted in the absence of any commercial or financial relationships that could be construed as a potential conflict of interest.

## Footnotes

^1^http://larssonlab.org/tcga-viruses/report_BRCA.php

^2^http://ccs.miami.edu/

^3^http://pave.niaid.nih.gov/

## Abbreviations

APOBEC3B, apolipoprotein B mRNA editing enzyme, catalytic polypeptide-like 3B; CIN, cervical intraepithelial neoplasia; EBV, Epstein–Barr virus; ER, estrogen receptors; HER 2 (also known as ErbB-2), human epidermal growth factor receptor 2 protein; HPV, human papilloma virus; HPV E7, human papilloma virus E7 protein; IHC, immunohistochemistry; NGS, next generation sequencing; PCR, polymerase chain reaction; PR, progesterone receptors; *r*, correlation co-efficient; TCGA, the cancer genome atlas.
