# Supplementary material for: Human Papilloma Viruses and Breast Cancer
Source: Front Oncol. 2015 Dec 16;5:277. doi: 10.3389/fonc.2015.00277 (PMC4679879; doi:10.3389/fonc.2015.00277)
Supplement: Supplementary file 1 [file data_sheet_1.docx]

**Supplementary Detailed Materials and Methods**

**Identification of HPV gene sequences by polymerase chain reaction (PCR).**

*In situ* PCR, semi-nested PCR and real time PCR were used for the detection of HPV. All PCR products were sequenced to help identify any contamination. Although *in situ* PCR can produce false positive outcomes, use of this method can add to the validity of results based on semi-nested and real time PCR.

*Preparation of genomic DNA*

For standard and real time PCR, the methods were as described in Steinau et al (19). The primers used were β-actin5’ *forward* (5’CTTCTGCCGTTTTCCGTAGG 3’) and β-actin3’ *reverse* (5’TGGGATGGGGAGTCTGTTCA3’) at the final concentration of 1 μM . Thermal cycles were: 94 º C for 15 minutes; 94 º C for 30 seconds, 55 º C for 30 seconds, 72 º C for 45 seconds for 30 cycles. HotStarTaq Master Mix Kit (QIAGEN) was used for the PCR reaction master mix. gDNA samples, which were β-actin positive, were selected for the detection of HPV genomes.

*Standard PCR*

The primers for semi-nested PCR My11 (5’GCACAGGGYCAYAAYAATGG3’) to modified GP6 (5’AATCATATTCCTCMMCATGTC3’). The second round was Gp5+ (5’TATTTGTTACTGTKGTWGATAC3’) to Gp6+. These primers were degenerate for HPV16 and 18, but were also capable of bringing up types 3, 11, 12, 45, 58, 73 and 75.

Stringent negative controls were used in parallel with all PCR analyses. These negative controls were no DNA (water) and a reagent blank, plus sequencing of the products of these controls in case the bands could not be seen on a gel. Negative outcomes of PCR analyses of selected breast cancer specimens provided negative controls based on breast tissues. The positive control for HPV was an HPV 18 positive HeLa cell line.

*Real-time PCR*

The HPV L1 gene in gDNA samples was amplified using a real-time PCR machine (Rotor

Gene Q, QIAGEN). Thermal cycles used were: 95 º C for 5 minutes, 95º C for 10 seconds and 60 º C for 30 seconds for 60 cycles. The PCR reaction mix was the Quanti Fast SYBR Green kit (QIAGEN) and was used according to the manufacturer’s recommendation. The primers used for detection of HPV were *GP05+ forward* (5’TTTGTTACTGTGGTAGATACTAC3’; and *GP06+ reverse* (5’AAATCATATTCCTCMMCATGTC3’) at the final concentration of 1 μM. The quantity of gDNA used for PCR was determined by estimating theoretical gene copies of HPV 16 in breast and breast cancer tissues. A positive control for HPV was a purified PCR product containing the HPV L1 region. It was amplified with MY11 and MY09 primers. Negative controls were (i) ultrapure H_2_O (*no DNA* *template*) and (ii) extraction blank (a DNA extraction control without formalin fixed paraffin embedded – FFPE, tissues).

The PCR products were analysed and selected for sequencing based on amplification profiles and the achievement of the known melting point. The analysis was performed using Rotor Gene Q software (QIAGEN).

Sequencing the PCR products and identification of HPV types

The HPV PCR products from GP5 to Gp6 were sequenced to determine the HPV type. The HPV genotypes were identified by BLAST via the US National Center for Biotechnology Information.

*In-situ* PCR

These analyses were conducted as described in Heng et al [10]. *In situ* PCR is less susceptible to contamination and has the important advantage of localizing the specific genetic material at the cellular level.

In-situ PCR incorporates a DIG labelled nucleotide into the product formed. This is detected by using an anti-DIG antibody followed by NBT/BCIP. (nitroblue tetrazolium salt + 1. 5-bromo-4-chloro-3-indolyl-phosphate) which yields a blue colour. The slide is then counterstained with eosin which is pink.

The identification by PCR of HPV in benign non - malignant breast or breast cancer specimens was considered as positive if two or more of the following outcomes in the same specimen were observed: (i) HPV DNA sequences identified by standard PCR and/or real time PCR, (ii) HPV positive *in situ* PCR.

*Confirmatory PCR studies*

DNA extracts from six selected (for positive HPV) benign and breast cancer specimens were independently analysed by the Antonsson group at the QIMR Berghofer Medical Research Institute, Brisbane, for the presence of HPV DNA using their previously published methods [20].

**HPV biological activity.**

An assessment of HPV biological and oncogenic activity was conducted by (i) the identification of HPV RNA sequences which are an indication of HPV transcription activity (ii) the identification of HPV E7 proteins, (iii) the high expression of p16 protein, (iv) high expression of ER, (v) inhibition of p53 protein expression, and (vi) the identification of HPV associated koilocytes.

Antibodies, that are specific for HPV E7 proteins, have recently been developed (23). These antibodies (Cervimax) were used in this study. The specificity of these antibodies has been demonstrated experimentally and by epidemiological studies [23]. The HPV E7 antibody reacts with a wide range of HPV types including high risk for cancer HPV 16 and 18. HPV E7 antibodies have not been previously used on breast tissues.

p16. p16 contributes to regulation of the cell cycle. There is substantial evidence which indicates that p16 expression is associated with HPV biological activity [24]. High expression of p16 as a surrogate for HPV E7 activity is a sound indication of transcriptionally active HPV [25]. In breast tissues negative or low expression of p16 is present in normal ductal epithelial tissues with a progressive increase of p16 expression in benign and malignant breast lesions [26-28]. The situation is complicated by Epstein Barr virus (EBV) which blocks the expression of p16 [29].

p53. The inhibition of expression of the cell death related p53 protein by HPV E6 protein is a well documented mechanism for HPV oncogenesis. p53 expression is inhibited in HPV 16 positive breast cancer [30].

ER. Estrogens have an essential role in breast cancer and high ER expression is a feature of most breast cancers. Recently, high ER expression has been associated with HPV positive breast cancers [9].

HER 2 (ErbB-2). The HER 2 receptor co-operates with HPV E6 and E7 oncoproteins in breast oncogenesis [33].

**Immunohistochemistry (IHC).**

Standard manual IHC methods were used with the omission of the antigen retrieval step for the identification of HPV E7 protein expression, The antibodies were: anti HPV E7 monoclonal “Cervimax” - Valdospan GmbH. Austria. Positive controls for the E7 antibody were the Hela (HPV18) cell line and cervical tissues that were positive by PCR and sequencing. Freshly cut slides and the cell lines needed less antibody (1/500 for 30 mins) than recommended by the manufacturer (Valdospan). Slides that were up to 5 years old needed 1/100 dilution of antibody for 1 hour. Good outcomes for HPV E7 were achieved with clear staining of both nuclei and cytoplasm of benign breast and breast cancer cells with 1 to 100 or 1 to 500 dilution of the antibody without antigen retrieval. The outcomes in the results section are based on staining of the nuclei.

IHC for estrogen receptor (ER), progesterone receptor (PR), and human epidermal growth factor receptor 2 (HER 2) were previously performed as part of the standard assessment of breast cancer specimens. For benign breast specimens the same assessments were conducted as part of this current project. Standard methods were used with a Benchmark XT autostainer

(Ventana Medical Systems Inc, Tucson, AZ) using the I-View detection kit. These and additional IHC analyses were conducted at Douglass Hanly Moir- Pathology in Sydney, Australia. The antibodies were as follows: ER, monoclonal antibody; 1:1 dilution, Ventana, catalog 76O-2596; PR, monoclonal antibody, 1:60 dilution, DAKO catalog M3569; P16 monoclonal antibody, Ventana CINtec, catalog 725-4713; anti- p53 monoclonal antibody, Abcam Pab 240; HER2/neu monoclonal antibody Ventana catalog 790-2991. For invasive breast cancer specimens silver fluorescence *in situ* hybridization analyses for HER-2 amplification was performed in addition to IHC using using the Path Vysion kit (Des- Plaines, IL).

ER, PR, p16 and p53 proteins are expressed in cell nuclei. HER 2 protein is expressed in cell cytoplasm and membranes. HPV types 16 and 18 E6 protein is expressed in both cell nuclei and cytoplasm. Anatomical pathologists from Douglass Hanly Moir – Pathology, conducted the routine assessment of ER, PR and HER in breast cancer specimens. Two independent observers (JL and WG) used the same methods for the assessment of ER, PR and HER in the benign breast specimens. The staining of breast tissues during immunohistochemistry procedures is heterogenous with staining frequently confined to only part of a tissue section. The assessments were based on those tissue sections with positive staining cells. The outcomes were assessed by the intensity of staining on a scale of 0 and 1 to 3. Positive (HPV-positive cervical cancer specimen) and negative (antibody omitted) controls were used for each batch of specimens.

In breast cancer specimens IHC staining for p16 appears to differ from the patterns commonly seen in cervical neoplasia. p16 expression in both non invasive and invasive breast cancer specimens are most commonly seen in clusters of cancer cells with no staining in other parts of the cancer specimen [25]. For this reason we have adopted the same methods of assessment as outlined above for ER and PR but confined to assessments of percentages of positive and intensity of staining cancer cells in the clusters. We have adopted the same approach to assessment of p53 which also commonly stains in clusters in breast cancer specimens.
